# Supplementary material for: The Evolution and Characterization of the RNA Interference Pathways in Lophotrochozoa
Source: Genome Biol Evol. 2024 May 7;16(5):evae098. doi: 10.1093/gbe/evae098 (PMC11114477; doi:10.1093/gbe/evae098)
Supplement: evae098_Supplementary_Data [file evae098_supplementary_data.zip › Supplementary_materials.zip!/selected_miRNAs/NoGe_selected_miRNAs/mirdeep_pdf_files/NMRB01001256.1_Notospermus_geniculatus_strain_Ushimado_scaffold1256,_whole_genome_shotgun_sequence_26752.pdf]

Provisional ID	:	NMRB01001256.1_Notospermus_geniculatus_strain_Ushimado_scaffold1256,_whole_genome_shotgun_sequence_26752
Score total	:	68.8
Score for star read(s)	:	3.9
Score for read counts	:	62.8
Score for mfe	:	2.7
Score for randfold	:	0
Score for cons. seed	:	-0.6
Total read count	:	135
Mature read count	:	36
Loop read count	:	0
Star read count	:	99

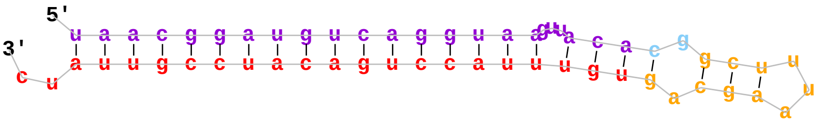

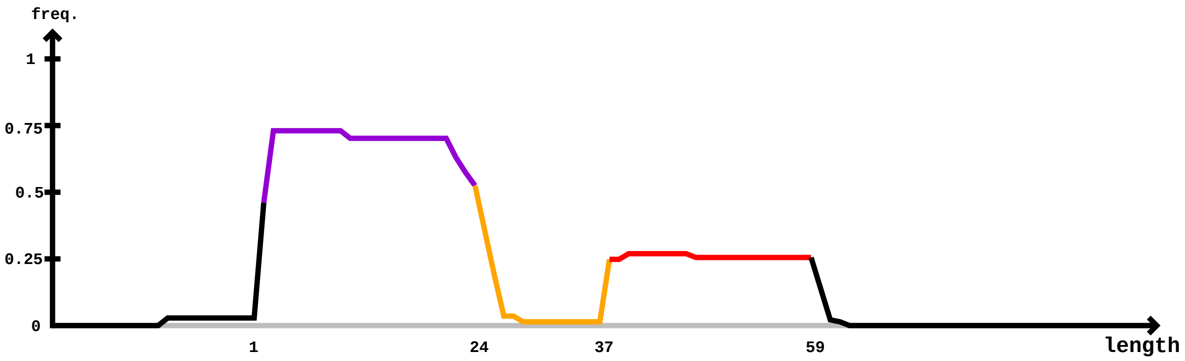


```
                    Star                              Mature
5'- aaaacguaucgcauaacggguaacggaugucagguaaguuacacggcuuuaagcaguguuuaccugacauccguuauccguuaugcgauacgacguuaucagucaaaacauug   -3'    obs
    aaaacguaucgcauaacggguaacggaugucagguaaguuacacggcuuuaagcaguguuuaccugacauccguuauccguuaugcgauacgacguuaucagucaaaacauug          exp
    .....((((((((((((((((((((((((((((((((...((((.(((...))).))))))))))))))))))))))))))))))))))))(((.......))).........    reads      mm      sample
    ........................................................uguuuaccugacauccguuaCc...................................    1          1       Mm2
    ..........................................................uuuaccugacauccguuaCccgu................................    1          1       Mm2
    ..........................................................uuuaccugacauccguuauccgu................................    1          0       Mm2

    ....................uaacggaugucGgguaaguu.........................................................................    1          1       Fm3
    ....................uaacggaugucagguaaguu.........................................................................    9          0       Fm3
    ....................uaacggaugucagguaaguua........................................................................    8          0       Fm3
    ....................uaacggaugucGgguaaguuac.......................................................................    4          1       Fm3
    ....................uaacggaugucagguGaguuac.......................................................................    3          1       Fm3
    ....................uaacggaugucagguaaguuacU......................................................................    5          1       Fm3
    ....................uaacggaugucGgguaaguuaca......................................................................    6          1       Fm3
    ....................uaGcggaugucagguaaguuaca......................................................................    3          1       Fm3
    ....................uaacggaugucagguaaguuaca......................................................................    18         0       Fm3
    ....................uaacggaugucagguGaguuacac.....................................................................    1          1       Fm3
    ....................uaacggaugucagguaaguuacaA.....................................................................    3          1       Fm3
    .....................aacggaugucagguaaguuaca......................................................................    6          0       Fm3
    .....................aacggGugucagguaaguuaca......................................................................    1          1       Fm3
    .....................aacggaugucGgguaaguuaca......................................................................    3          1       Fm3
    .....................aGcggaugucagguaaguuacac.....................................................................    1          1       Fm3
    .....................aacggaugucGgguaaguuacacg....................................................................    1          1       Fm3
    .....................aGcggaugucagguaaguuacacg....................................................................    21         1       Fm3
    .....................aacggaugucagguaaguuacacggc..................................................................    5          0       Fm3
    ...............................................Cuuaagcaguguuuaccu................................................    2          1       Fm3
    ........................................................uguuuaccugacauccguuauc...................................    30         0       Fm3
    ........................................................uguuuaccugacauccgAuauc...................................    1          1       Fm3
    ........................................................uguuuaccugacauccgCuauc...................................    1          1       Fm3
    ..........................................................uuuaccugacauccguuauccg.................................    1          0       Fm3

    ..........gcauaacggAuaacggaug....................................................................................    4          1       Fm2
```
